# Combined Two-Photon Excitation and d→f Energy Transfer in a Water-Soluble Ir^III^/Eu^III^ Dyad: Two Luminescence Components from One Molecule for Cellular Imaging

**DOI:** 10.1002/chem.201403618

**Published:** 2014-06-16

**Authors:** Elizabeth Baggaley, Deng-Ke Cao, Daniel Sykes, Stanley W Botchway, Julia A Weinstein, Michael D Ward

**Affiliations:** [a] Department of Chemistry, University of SheffieldSheffield S3 7HF (UK); [b] State Key Laboratory of Coordination Chemistry, School of Chemistry and Chemical Engineering, Nanjing UniversityNanjing 210093 (P.R. China); [c] Rutherford Appleton Laboratory, STFC, Research Complex at Harwell, Harwell Science and Innovation CampusDidcot OX11 0FA (UK)

**Keywords:** energy transfer, europium, imaging agents, iridium, luminescence

## Abstract

The first example of cell imaging using two independent emission components from a dinuclear d/f complex is reported. A water-stable, cell-permeable Ir^III^/Eu^III^ dyad undergoes partial Ir→Eu energy transfer following two-photon excitation of the Ir unit at 780 nm. Excitation in the near-IR region generated simultaneously green Ir-based emission and red Eu-based emission from the same probe. The orders-of-magnitude difference in their timescales (Ir ca. μs; Eu ca. 0.5 ms) allowed them to be identified by time-gated detection. Phosphorescence lifetime imaging microscopy (PLIM) allowed the lifetime of the Ir-based emission to be measured in different parts of the cell. At the same time, the cells are simultaneously imaged by using the Eu-based emission component at longer timescales. This new approach to cellular imaging by using dual d/f emitters should therefore enable autofluorescence-free sensing of two different analytes, independently, simultaneously and in the same regions of a cell.

The use of luminescent molecules as probes for cellular imaging is an area of immense interest. The high sensitivity and high spatial and temporal resolution associated with fluorescence microscopy provide possibilities for non-invasive monitoring of processes over length scales ranging from single-molecule interactions to whole organisms.[1] The use of two-photon excitation provides high spatial resolution[2] and, together with fluorescence lifetime imaging microscopy (FLIM),[3] has shown how the lifetime domain can provide information which is complementary to that obtained by using steady-state intensity-based imaging. FLIM is an excellent method to probe the environment of a molecule, because excited-state lifetimes are sensitive to environmental changes such as pH, viscosity, oxygen concentration and refractive index. There has been tremendous success in developing and using fluorescent probes with new characteristics in FLIM.[4] However, disadvantages of current standard fluorescent probes with short emission lifetimes are that 1) they emit on the same timescale as background autofluorescence; and 2) the variations in lifetime required for FLIM purposes are relatively small.

Phosphorescent metal complexes with long-lived triplet excited states are therefore of particular interest,[5] because they offer substantial advantages compared to the more traditional organic fluorophores.[1] These advantages include tunability of absorption and emission maxima over a wide range by using simple ligand substitutions, and long-lived (>10^−7^ s) luminescence, which allows rejection of short-lived autofluorescence and also provides larger, easier to detect variations in emission lifetime. Examples of cell imaging by using steady-state emission from metal-based luminophores[5–10] include complexes of Ru^II^,[6] Re^I^,[7] Ir^III^,[8] Pt^II[9]^ and lanthanides,[10] using both one- and two-photon excitation. However, there are few reports of cellular imaging using variations in the lifetime of metal-complex phosphorescence, known either as time-resolved emission microscopy (TREM)[9a, 12] or phosphorescence lifetime imaging microscopy (PLIM).[9a, 11] Furthermore, it has only recently become possible to perform such studies using two-photon excitation, which permits essential high spatial resolution (submicrons) and enables the use of tissue-friendly near-IR excitation.[11d]

Herein, we report a new approach to cellular imaging by using dinuclear d/f metal complexes with two luminescence outputs, in different spectral regions, and with emission lifetimes which differ by three orders of magnitude. This is the first example of applying the new method of two-photon PLIM[11d] to imaging using lanthanide complexes, and the first example of exploiting d→f energy transfer in live-cell imaging. We[12,13] and others[12,14] have previously studied heterometallic d/f complexes in detail, showing that phosphorescent d-block units can act as effective sensitizers of luminescent excited states of lanthanide ions by a range of energy-transfer mechanisms.[13] The d→f energy transfer in these dyads is often incomplete because of poor donor/acceptor spectral overlap.[12] This results in sensitisation of luminescence from the f-block component, whilst not completely quenching luminescence of the d-block component, such that emission occurs from both metal centres by using a single-excitation wavelength. This method is conceptually related to that reported recently by Yoshihara et al., who reported dual luminescence from a complex containing coumarin and Ir-based luminophore units as the basis of a ratiometric luminescent oxygen sensor.[15]

The prototype is the Ir^III^/Eu^III^ dyad **1⋅Eu**, in which emission from both metal centres occurs following excitation of only the Ir^III^ unit and subsequent partial Ir^III^→Ln^III^ energy transfer (Scheme 1). The appreciable two-photon absorption cross-section of the Ir^III^ unit[16] means that both visible-region luminescence components could be generated by excitation in the near-IR region.[17] The “control” complex **1⋅Gd** contains the same Ir-based unit but has no lanthanide-based luminescence (we note that related d/f complexes, which combine a phosphorescent d-block unit with a Gd^III^ centre, have been of interest for combining two imaging modalities—luminescence+MRI—with a single-probe molecule).[18]

**Scheme 1:**
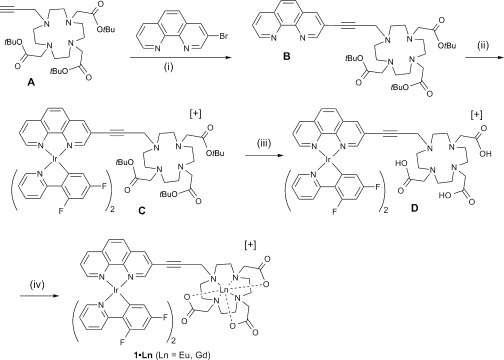
Synthesis of the complexes 1⋅Eu and 1⋅Gd. i) [PdCl_2_(PPh_3_)_2_], CuI, Et_3_N, MeCN; (ii) [IrCl(F_2_ppy)_2_]_2_, CH_2_Cl_2_/MeOH; (iii) CF_3_CO_2_H/CH_2_Cl_2_; (iv) Ln(CF_3_SO_3_), water (pH 6.5).

Compounds **1⋅Eu** and **1⋅Gd** contain an [Ir(F_2_ppy)_2_(phen)]^+^ chromophore (F_2_ppy=anion of 2-(2,4-difluorophenyl)pyridine; phen=1,10-phenanthroline), which showed characteristic luminescence in the green region.[19] The excited-state energy of this Ir^III^ chromophore, following either one-photon[13] or two-photon[17] excitation, is sufficient to sensitise the emissive ^5^D_0_ excited state of Eu^III^. The pendant aminocarboxylate macrocycle will provide high kinetic and thermodynamic stability to the lanthanide ion in water, in contrast to the Ir/Eu dyads that we have studied previously, which were only stable in non-competitive solvents, such as CH_2_Cl_2_.[13,17]

The syntheses of the complexes is summarised in Scheme 1 (full details are given in Supporting Information). The key step is the Pd-catalysed coupling of the propargyl-substituted cyclam derivative **A** with 3-bromo-phenanthroline to give **B**, which contains the phenanthroline binding site for the Ir^III^ unit and the protected aminocarboxylate macrocycle for the Ln^III^ ion. Coordination of the {Ir(F_2_ppy)_2_}^+^ unit to the phen site (**C**), ester hydrolysis to liberate the aminocarboxylate ligand (**D**) and finally reaction with the relevant lanthanide triflate salts to give **1⋅Eu** and **1⋅Gd** all followed standard methods.

UV/Vis spectra of **1⋅Eu** and **1⋅Gd** in water (Figure 1 a) showed the expected aromatic ligand-centred π-π* transitions in the UV region and transitions associated with the Ir^III^ chromophore at lower energy (a clear feature at ca. 360 nm with an absorption tail in the 400–500 nm region), allowing one-photon excitation with visible light, or two-photon excitation in the near-IR region.

**Figure 1:**
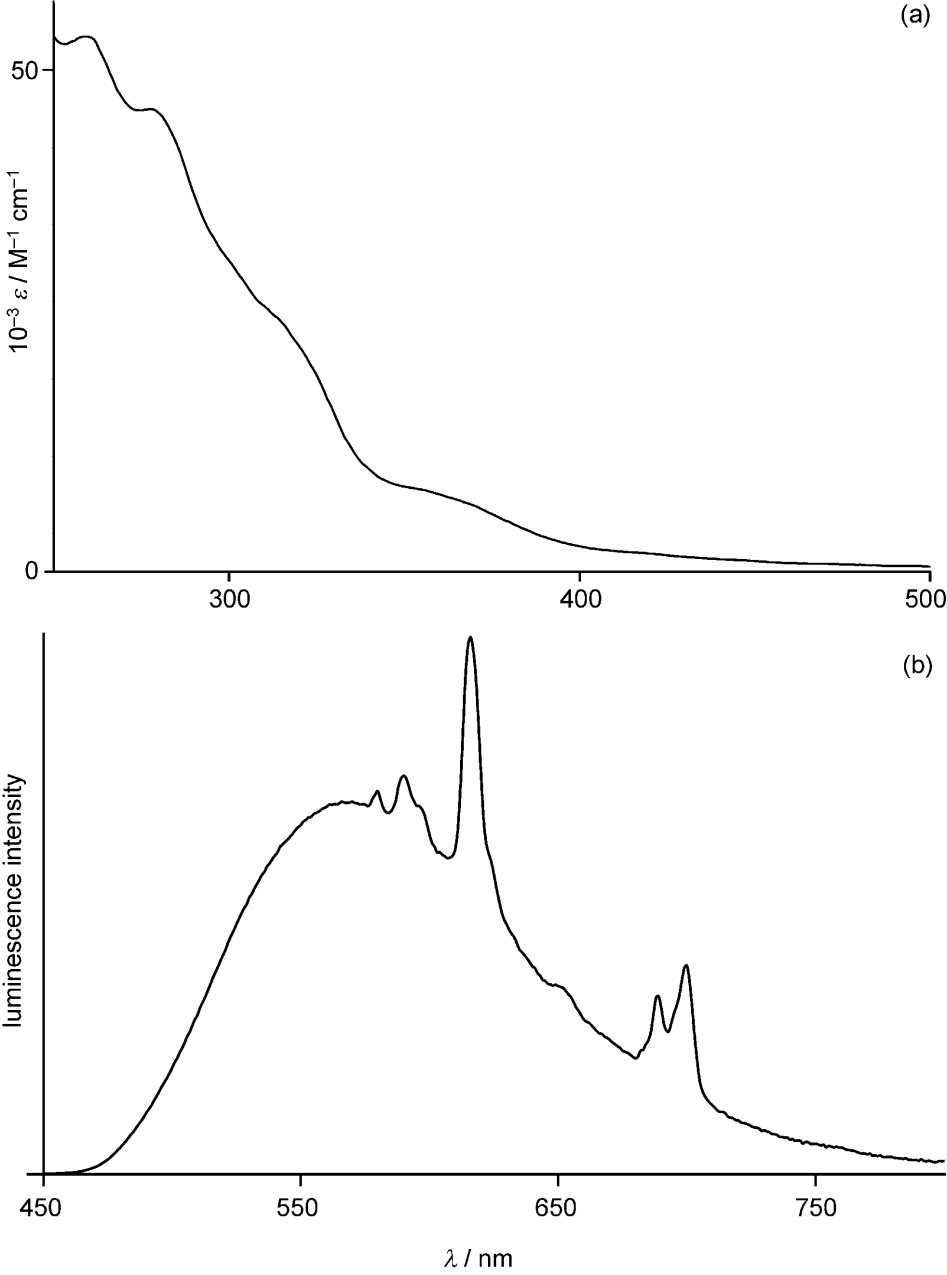
a) UV/Vis absorption spectrum and b) luminescence spectrum (*λ*_ex_=400 nm) of 1⋅Eu in water.

The steady-state emission spectrum (*λ*_exc_=400 nm) of **1⋅Gd** showed a broad and unstructured emission maximum at 555 nm, characteristic of a dominant Ir→phen ^3^MLCT excited state,[19] with *t*=410 ns and quantum yield *ϕ*=0.23 in aerated water. In agreement with the ^3^MLCT assignment, the rigidochromism is substantial; at 77 K the emission maximum blueshifts to 476 nm giving an excited-state energy of 21 000 cm^−1^, more than sufficient for sensitisation of the luminescent ^5^D_0_ state of Eu^III^ at approximately 17 500 cm^−1^. In the emission spectrum of **1⋅Eu**, strong sensitised Eu-based emission with the characteristic sharp lines from the ^5^D_0_→^7^D_n_ manifold superimposed on the Ir-based emission profile can be also seen (Figure 1 b). The lifetime of the Ir-based emission in **1⋅Eu** is reduced to 350 ns, giving an Ir^III^→Eu^III^ energy-transfer rate constant *k*_EnT_ of approximately 4×10^5^ s^−1^ based on Equation (1) (in which *τ*_u_ and *τ*_q_ are the “unquenched” and “quenched” Ir-based emission lifetimes of **1⋅Gd** and **1⋅Eu**, respectively):

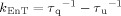
(1)

The Ir-based luminescence decay of **1⋅Eu** in deoxygenated water showed two components with lifetimes of 1070 and 380 ns (major and minor components, respectively). The presence of two components (in particular the minor one) may arise from aggregation effects, as was observed previously in related Ir complexes,[13d] with the major component being the monomer luminescence under deoxygenated conditions and the minor component arising from partially quenched aggregates. The sensitised Eu-based emission lifetime is 0.46 ms in H_2_O and 1.35 ms in D_2_O, giving a value for number of coordinated water molecules, *q*, of one,[20] which is reasonable given that the ligand is heptadentate to Eu^III^. A quantum yield for the Eu-based emission could not be measured directly due to its overlap with the residual Ir-based emission, but from the lifetime of 0.46 ms in H_2_O, we can estimate a quantum yield of approximately 0.15.[21,22]

To evaluate the feasibility of using the two different luminescence components from **1⋅Eu** for imaging, we measured the photophysical properties of the complexes in cells using both one- and two-photon excitation. Successful uptake of **1⋅Eu** into living cells was achieved by using either phosphate-buffered saline (PBS; 10 min at 50 μm, 0.25 % DMSO) or Dulbecco’s modified Eagle’s medium (DMEM; 4 h, 10–100 μm, 0.04–0.4 % DMSO) as the incubation medium (see the Supporting Information). Emission spectra measured from live cells pre-treated with **1⋅Eu** and **1⋅Gd** presented in Figure 2 showed no major changes compared to the spectra measured in aqueous solution, apart from a small blueshift of the Ir-based emission maximum, which can be ascribed to the solvatochromic (hence, environment-dependent) nature of the ^3^MLCT excited state.[19] Imaging by using the Ir-based emission component from either **1⋅Eu** or **1⋅Gd**—under two-photon excitation, as well as one-photon excitation—was possible, because the two-photon absorption cross-section of the Ir^III^ chromophore was found to be approximately 13 GM at 760 nm (see the Supporting Information).

**Figure 2:**
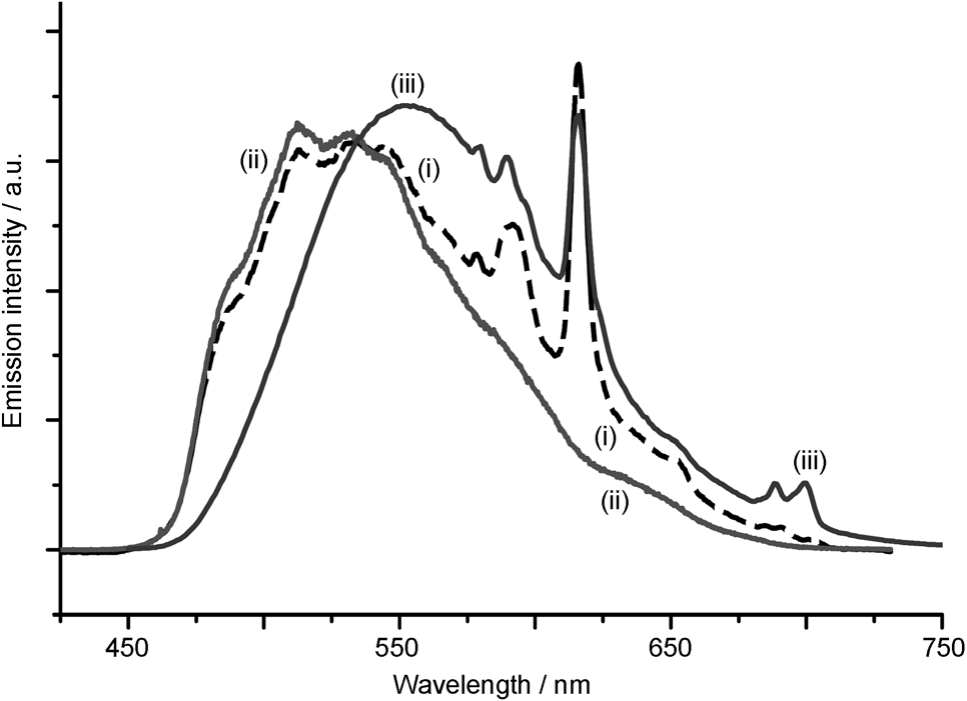
Two-photon (*λ*_ex_=780 nm) emission spectra taken directly from live CHO cells pre-treated with 1⋅Eu [trace (i), dashed line] and 1⋅Gd [trace (ii)]. The single-photon (400 nm) emission spectrum of 1⋅Eu in H_2_O [trace (iii)] is included for comparison.

A typical steady-state confocal microscopy image of **1⋅Eu** in live human dermal fibroblast (HDF) cells under 760 nm excitation (Figure 3 a and b) revealed both punctate and diffuse cytoplasmic staining, which became more localised towards the perinuclear region—similar to what has been observed for other Ln^III^ and Ir^III^-based complexes used in cell imaging.[5d, g, 23] Furthermore, time-resolved imaging of **1⋅Eu**-labelled HDF cells by using a Becker and Hickl combined FLIM/PLIM imaging unit, which uses a time-correlated single-photon counting module to record emission decays from each pixel of a 256×256 array, showed how the Ir-based emission lifetime remains approximately constant across different cellular locations (Figure 3 c, d, e). Decay kinetics taken from individual pixels and larger regions of interest (Figures 3 d, e, and S2 in the Supporting Information), which clearly demonstrated consistent Ir-based emission decay in different regions of the cell, are best fit to a double exponential model with Ir-based emission lifetimes of 1040±80 and 235±80 ns. These values are similar to those recorded in deoxygenated solution, implying that when **1⋅Eu** is taken up into the cells, the Ir unit is protected from being quenched by molecular O_2_ within the cell.

**Figure 3:**
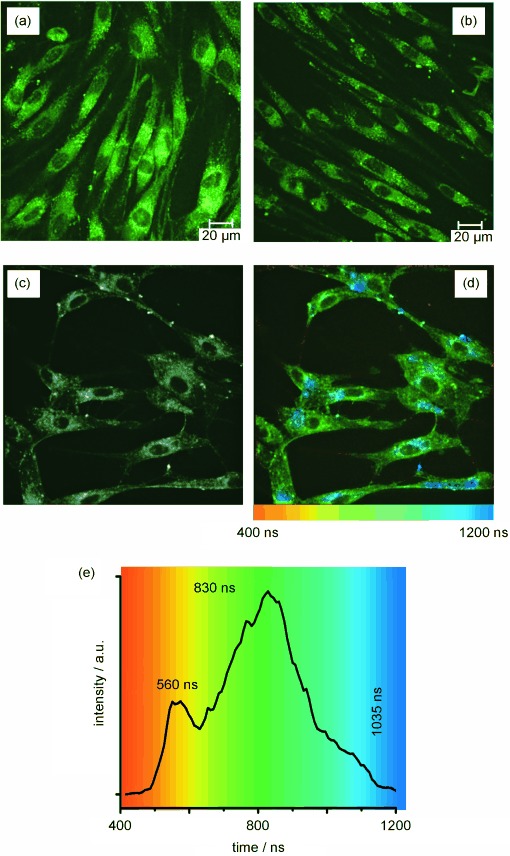
Two-photon (*λ*_ex_=780 nm) confocal (a, b) and PLIM (c–e) imaging of live HDF cells labelled with 1⋅Eu. a), b) Steady state confocal microscopy images (*λ*_em_=500–550 nm) using cells incubated with 1⋅Eu at 100 and 50 μm, respectively. c)–e) PLIM images as follows: c) black and white intensity image (all emitted photons binned into a single channel); d) lifetime map showing Ir-based emission lifetime across cells; and e) overlaid emission decay curves and lifetimes of the major emission component from cellular locations 1–4.

When using PBS as the incubation medium for **1⋅Eu**, we noted that a small population of cells also exhibited emission from the cell nuclei (Figure 4 a, b). The Ir-based emission decay from **1⋅Eu** in the cell nuclei is similar to that observed in the cell cytosol and perinuclear region, with the main luminescence component being approximately 1100 ns (Figures 4 c, d and Figure S3 in the Supporting Information). Comparable images from cells labelled with **1⋅Gd** are presented in Figure S3 in the Supporting Information. Cellular Ir-based emission lifetimes for **1⋅Gd** are slightly longer with respect to **1⋅Eu** (nucleus *τ*=1300±65 ns; perinuclear region *τ*=1300±105 ns), consistent with lack of internal energy-transfer quenching of the Ir chromophore.

**Figure 4:**
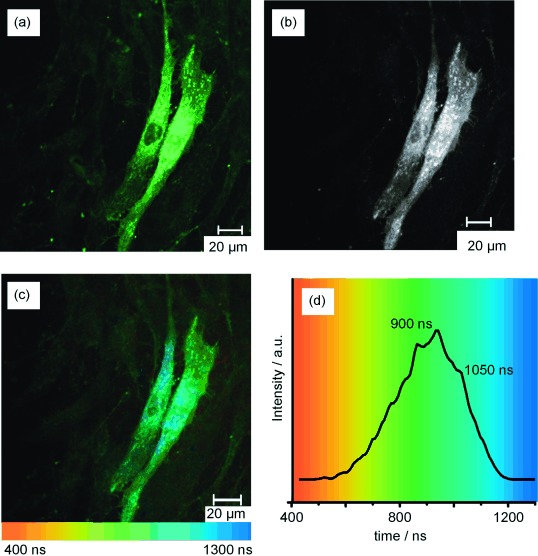
Two-photon (*λ*_ex_=780 nm) confocal a) and PLIM b)–d) imaging of live HDF cells labelled with 1⋅Eu (50 μm, 0.25 % DMSO in PBS). a) Steady-state confocal microscopy images (*λ*_em_=500–550 nm). b)–d) PLIM images as follows: b) black and white intensity image (all emitted photons binned into a single channel); c) lifetime map showing variations of Ir-based emission lifetime across cells; and d) overlaid emission decay curves and lifetimes of major emission component from cellular locations 1–3.

A co-staining experiment using **1⋅Eu** and propidium iodide (PI), a nuclear cell stain which cannot permeate the membrane of living cells, revealed that cells exhibiting additional nuclear Ir-based emission also showed positive PI staining (Figure S5 in the Supporting Information); the same was observed with **1⋅Gd**. Positive co-localisation between PI and **1⋅Ln** suggest that the membranes of these cells may be compromised, and are therefore likely to be in the early stages of cell death. 3-(4,5-Dimethylthiazol-2-yl)-2,5-diphenyltetrazolium bromide (MTT) toxicity data also revealed a slight reduction in cell metabolic activity when PBS was used as the incubation medium (at 50 μm of **1⋅Ln**), supporting the positive PI staining result. Conversely, **1⋅Ln** incubations (10–100 μm) with DMEM showed no reduction in metabolic activity with respect to untreated cells (Figure S6 in the Supporting Information), so incubation with DMEM appeared to be preferable for imaging studies with this class of compound.

In addition to the Ir^III^-based imaging, the long-lived sensitised Eu-based luminescence from **1⋅Eu**, following two-photon excitation of the Ir chromophore at 780 nm, also permitted images to be obtained by time gating the detection to reject the (relatively) short-lived Ir component. Figure 5 (left column) shows a series of two-photon induced time-gated images from **1⋅Eu** in HDF cells comprising a) all luminescence over the 0–100 μs window; and in b)–d) the total luminescence emitted after an initial delay of 20, 50 and 75 μs, respectively. Comparison with the same set of measurements from **1⋅Gd** (right column) clearly shows that the Ir-based emission has mostly decayed by 20 μs and is no longer visible after 50 μs. In contrast, all time-gated images recorded by using **1⋅Eu** exhibited sufficient emission to image the cell clearly; the cell structure remained visible even when selecting the longest time delay with the lowest number of photon counts (75–100 μs, trace (d)). This can only be from the sensitised Eu-based luminescence, which persists over hundreds of μs, because this long-lived emission is completely absent in cells labelled with **1⋅Gd**. Emission decay traces from **1⋅Eu**-labelled HDF cells recorded by using this extended 100 μs PLIM imaging window revealed the longest emission component to have a decay lifetime consistent with the observed Eu-based decay (0.46 ms in water, see above). Thus, cells treated with **1⋅Eu** can be successfully imaged using either the d- or f-emission component independently, with selection between the two based on time-gated detection.

**Figure 5:**
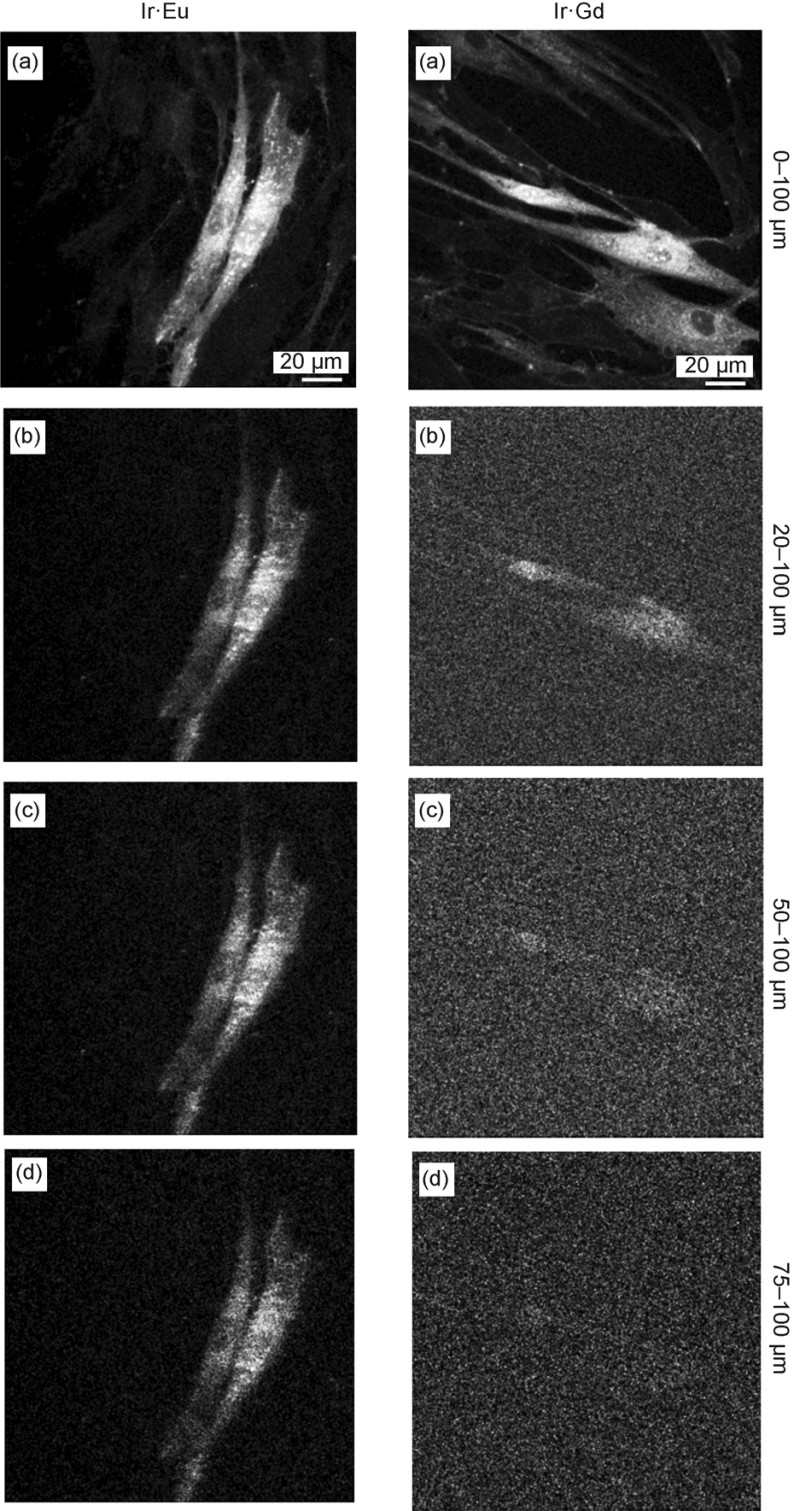
Two-photon (*λ*_ex_=780 nm) PLIM time-gated images of HDF cells labelled with 1⋅Eu (left) and 1⋅Gd (right) at 50 μm (0.25 % DMSO in PBS, 10 min at 37 °C). Images show: a) all emitted photons (0–100 μs); photons emitted during the time intervals following excitation of b) 20–100 μs; c) 50–100 μs; and d) 75–100 μs.

In conclusion, these stable and water-soluble d–f complexes demonstrated several new features of benefit for imaging purposes. Firstly, partial d→f energy transfer following two-photon excitation of the Ir^III^ unit allowed near-IR excitation to generate both Ir-based (μs timescale) and Eu-based (ms timescale) emission components, which can be used independently as the basis of cellular imaging using time-gated detection. Secondly, the Ir-based emission allows autofluorescence-free, time-resolved imaging and lifetime mapping in cellulo under two-photon excitation, with submicron spatial resolution, on a timescale orders of magnitude longer than fluorescence. This combined d–f approach to cellular imaging should therefore enable autofluorescence-free sensing of two different analytes, independently, simultaneously and in the same regions of a cell, without the necessity for co-localisation of two different probes and multiple excitation wavelengths.
