# Supplementary material for: Combined Two-Photon Excitation and d→f Energy Transfer in a Water-Soluble IrIII/EuIII Dyad: Two Luminescence Components from One Molecule for Cellular Imaging
Source: Chemistry. 2014 Jun 16;20(29):8898–903. doi: 10.1002/chem.201403618 (PMC4145666; doi:10.1002/chem.201403618)
Supplement: Supplementary file 1 [file chem0020-8898-sd1.pdf]

CHEMISTRY
A European Journal

Supporting Information

© Copyright Wiley-VCH Verlag GmbH & Co. KGaA, 69451 Weinheim, 2014

Combined Two-Photon Excitation and d$\rightarrow$f Energy Transfer in a Water-Soluble Ir^{III}/Eu^{III} Dyad: Two Luminescence Components from One Molecule for Cellular Imaging

Elizabeth Baggaley,[a] Deng-Ke Cao,[a, b] Daniel Sykes,[a] Stanley W. Botchway,[c] Julia A. Weinstein,*[a] and Michael D. Ward*[a]

chem_201403618_sm_miscellaneous_information.pdf

Supporting information

Part 1: Synthesis and characterisation – experimental data

Part 2: Cell culture and staining

Part 3: Cell imaging and photophysical measurements

Part 4: Additional references

Part 5: Figures S2 – S7

Part 1: Synthesis and characterisation – experimental data

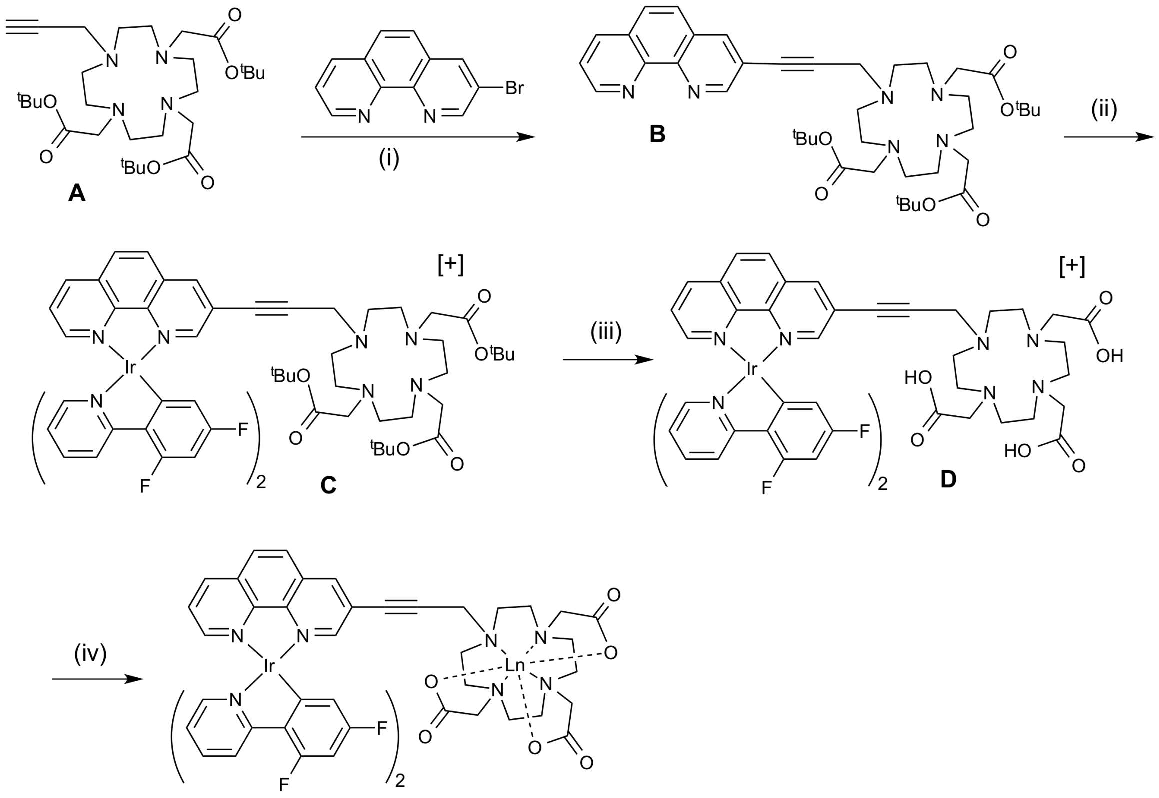

1•Ln (Ln = Eu, Gd)

Scheme 1. Synthesis of the complexes **1•Eu** and **1•Gd**, and the precursors **A – D**. (i) $Pd(PPh_3)_2Cl_2$, CuI, Et_3N, MeCN; (ii) $[Ir(dfppy)_2Cl]_2$, CH_2Cl_2/MeOH; (iii) CF_3CO_2H / CH_2Cl_2; (iv) $Ln(CF_3SO_3)$, water (pH 6.5).

Materials and Physical Measurements

Compounds 3-bromo-phenanthroline,[S1] 4,7-Bis-*tert*-butoxycarbonylmethyl-10-prop-2-ynyl-1,4,7,10-tetraaza-cyclododec-1-yl)-acetic acid *tert*-butyl ester (**A**),[S2] and $[Ir(dfppy)_2Cl]_2$,[S3] were prepared according to literature methods. All other chemicals were obtained from commercial sources and used without further purification. ^1H NMR spectra were recorded on a Bruker AV-III 400 MHz instrument. Electrospray mass spectrometry (ES-MS) spectra were recorded using a Micromass LCT instrument. Elemental analyses were performed on a Perkin Elmer 2400 CHNS/O Series II elemental analyzer. UV-Vis absorption spectra were measured on a Cary 50 spectrophotometer, and luminescence spectra on a Jobin-Yvon Fluoromax 4 fluorimeter, at room temperature. Ir-based emission lifetimes were measured using the time-correlated single-photon- counting technique with an Edinburgh Instruments "Mini-τ" luminescence lifetime spectrometer, equipped with a 410 nm pulsed diode laser as an excitation source and a Hamamatsu-H5773-03 photomultiplier tube (PMT)

detector. The lifetimes were calculated from the measured data using the supplied software.

Synthesis of B

A mixture of 3-bromo-phenanthroline (1.0 mmol, 0.258 g), compound **A** (1.2 mmol, 0.663 g), $[Pd(PPh_3)_2Cl_2]$ (0.05 mmol, 0.035 g), CuI (0.06 mmol, 0.011 g), and dry triethylamine (1 cm^3) in CH_3CN (30 cm^3) was heated at reflux under N_2 for 5 days. After evaporation under vacuum, the brown residue was dissolved in CH_2Cl_2 and washed with aqueous KCN solution and water, respectively. The CH_2Cl_2 solution was dried with $MgSO_4$, and then was filtered. After evaporation of solvent, the resultant solid was purified by column chromatography on Al_2O_3 using CH_2Cl_2/CH_3OH (97/3, v/v), to give **B** as a brown solid (yield: 0.52 g, 71%). ^1H NMR (400 MHz, $CDCl_3$): δ (ppm) 1.37 (s, 18 H, 2 x *t*-Bu), 1.42 (s, 9H, *t*-Bu) 2.19-3.66 (br m, 24 H, 4 x NCH_2 and 4 x NCH_2CH_2N), 7.66 (dd, 1H, phen H^8), 7.76 (d, 1H, phen H^5 or H^6), 7.85 (d, 1H, phen H^6 or H^5), 8.28 (dd, 1H, phen H^7), 8.33 (d, 1H, phen H^4), 9.12 (1H, d, phen H^2), 9.19 (dd, 1H, phen H^9). ES-MS (CH_3CN): found m/z = 731.5 for $[M + H]^+$. Anal. found (calcd) for $C_{41}H_{58}N_6O_6{\cdot}7H_2O$: C, 57.0 (57.4); H, 7.9 (8.40); N, 9.6 (9.8)%.

Synthesis of $[Ir(dfppy)_2(B)][PF_6]$ (complex C)

A mixture of **B** (0.5 mmol, 0.365 g) and $[Ir(dfppy)_2Cl]_2$ (0.25 mmol, 0.305 g,) in CH_2Cl_2/CH_3OH solution (60 cm^3; 3/1, v/v) was stirred under N_2 at 50 °C for one day. After evaporation of solvents, the residue was mixed with CH_2Cl_2 (60 cm^3) and saturated KPF_6 aqueous solution (30 mL), and the mixture was vigorously stirred for 2 hours at room temperature. The CH_2Cl_2 layer was separated, dried with $MgSO_4$, filtered, and the solvents evaporated. The resultant solid was purified by column chromatography on Al_2O_3 using CH_2Cl_2/CH_3OH (100/0.5, v/v) as eluent. Complex **C** was isolated as a yellow solid (yield: 0.51 g, 70%). ^1H NMR (400 MHz, $CDCl_3$): δ (ppm) 1.44 (s, 9H, *t*-Bu), 1.47 (s, 18H, 2 x *t*-Bu), 2.31-3.73 (br m, 24H, 4 x NCH_2 and 4 x NCH_2CH_2N), 5.75 (dd, 1H), 5.82 (dd, 1H), 6.62 (m, 2H), 7.03 (t, 1H), 7.07 (t, 1H), 7.32 (d, 1H), 7.40 (d, 1H), 7.78 (m, 2H), 7.88 (dd, 1H), 8.15 – 8.36 (m, 6H), 8.68 (dd, 1H), 8.86 (d, 1H). ES-MS (CH_3OH): m/z = 1303.5 for $[M - PF_6]^+$, with the correct isotope pattern for Ir. Anal. found (calcd) for $C_{63}H_{70}F_4N_8O_6IrPF_6{\bullet}KPF_6{\bullet}H_2O$ C, 46.2 (45.8); H, 4.8 (4.4); N, 6.7 (6.8)%.

Synthesis of complex D

A mixture of complex **C** (0.16 mmol, 0.27 g), CH_2Cl_2 (5 cm^3) and trifluoroacetic acid (5 cm^3) was stirred at room temperature for one day. The solvents were removed under vacuum, and then the residue was washed with CH_2Cl_2 (3 x 10 cm^3) and CH_3OH (3 x 10

cm^3). The resultant solid was dissolved in the minimum amount of ethanol, and was then re-precipitated by addition of diethyl ether affording **D** as a yellow powder (yield: 0.20 g, 95%). ^{1}H NMR (400 MHz, CD_3OD): δ (ppm) 3 – 4 ppm, very broad set of overlapping signals with maxima at 3.08, 3.31, 3.48, 3.75, and 3.99 ppm (m, 24H, 4 x NCH_2 and 4 x NCH_2CH_2N). Similarly the aromatic signals were significantly broadened with no couplings resolved, likely due to aggregation in solution and to the slow exchange between conformations of the macrocyclic ring. In the aromatic region: δ (ppm) 5.81 (2H), 6.77 (2H), 7.02 (2H), 7.50 (1H), 7.64 (1H), 7.93 (3H), 8.1 – 8.2 (6H), 8.84 (1H), 9.03 (1H), all broad. ES-MS (CH_3OH) found m/z = 1135.3 for $[M - PF_6]^+$. Anal. found (calcd) for $C_{51}H_{46}F_4N_8O_6IrPF_6$: C, 47.7 (47.9); H, 4.1 (3.6); N, 8.4 (8.8)%.

Syntheses of complexes 1•Eu and 1•Gd

A mixture of **D** (0.015 mmol, 0.0192 g) and $Eu(CF_3SO_3)_3$ (0.016 mmol, 0.0095 g) in H_2O (3 cm^3) was stirred at room temperature. The pH value of the solution was adjusted with 0.05 M NaOH to about 6.5 every 8 – 16 hours until no further change was observed (typically 4 – 5 days). After evaporation under vacuum, the residue was purified by reverse-phase HPLC using a CH_3CN/H_2O mixture containing 0.1% formic acid as eluent in which the proportion of CH_3CN was gradually increased from 5% to 95%. The product was isolated as a yellow crystalline solid was obtained (Yield: 14 mg, 65%). ES-MS (CH_3OH): m/z = 1283.2 for $[M - PF_6]^+$ (calc. 1284.1). Anal. found (calcd) for $C_{51}H_{43}N_8O_6F_4EuIrPF_6 \bullet H_2O$: C, 42.8 (42.3); H, 3.5 (3.1); N, 7.5 (7.7)%.

Complex **1•Gd** was prepared in exactly the same way, using $Gd(CF_3SO_3)_3$ in place of $Eu(CF_3SO_3)_3$ (Yield: 14 mg, 58%). ES-MS (CH_3OH): m/z = 1288.2 for for $[M - PF_6]^+$, (calculated 1289.4). Anal. found (calcd) for $C_{51}H_{43}N_8O_6F_4EuIrPF_6 \bullet 4H_2O$: C, 40.9 (40.6); H, 3.7 (3.4); N, 7.1 (7.4)%.

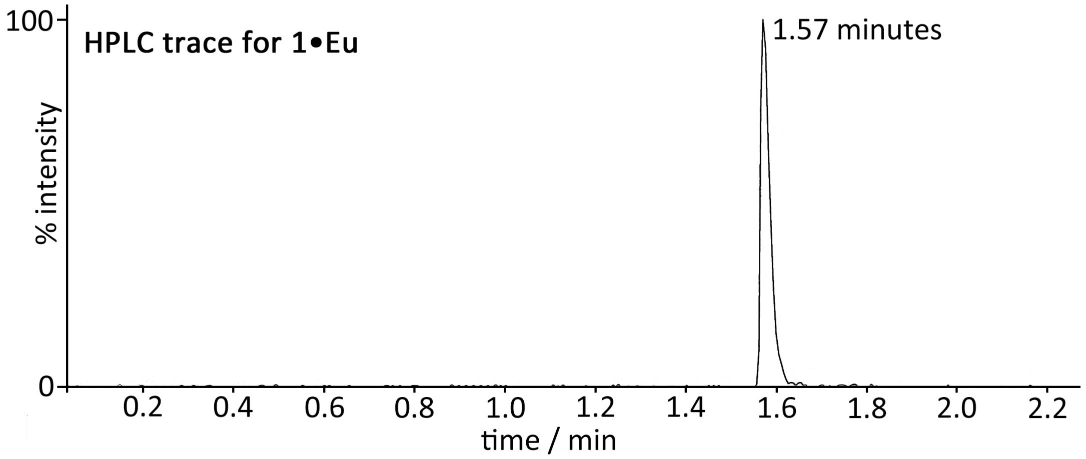

Fig. S1. HPLC trace for 1•Eu (see text above for conditions)

Table S1. UV-vis absorption bands of **1•Ln** at room temperature.

Compound	absorption bands (nm)
**1•Eu**	241, 259, 277, 310, broad band in the range of 320-450 nm
**1•Gd**	241, 259, 277, 310, broad band in the range of 320-450 nm

Table S2 Emissions and lifetimes of **1•Ln** in D_2O at room temperature.

Compound	Emission λ_{max} (nm)	Lifetime τ (ns)
**1•Eu**	569*, 580, 590, 616, 649, 689, 700	485
**1•Gd**	568*	530

* = Ir-based emission component.

Part 2: Cell culture and staining

(i) Cell cultures

Human Dermal Fibroblast (HDF) and Chinese Hamster Ovary (CHO) cells were cultured in a humidified 37°C, 5% CO_2/95% air (v/v) environment in Dulbecco's Modified Eagle's Medium (DMEM, Sigma-Aldrich) supplemented with 10% (v/v) FCS (fetal calf serum), L-glutamine (200 mM, 5 mL), penicillin/streptomycin (5 mL), and fungizone (1.25 ml). Both cell lines were cultured as monolayers in T-75 flasks and passaged using trypsin-EDTA. For PLIM and confocal imaging experiments, cells were seeded in to sterile 6 well plates and cultured until 60% confluent. For live cell emission spectra, cells were cultured in 35mm MatTek glass-bottomed dishes until 60% confluent.

(ii) Cell staining and imaging

After removal of growth media, cells were washed with PBS (phosphate buffered saline, 1 ml/well) before treating with a solution of the appropriate **1•Ln** complex: (i) 50 μM in PBS (0.25% DMSO, 10 minutes at 37°C, 1ml/well) or (ii) 10-100 μM in full DMEM (0.04-0.4% DMSO, 4h at 37°C, 1ml/well). All incubation solutions were diluted from a 20 mM stock solution of **1•Ln** in DMSO. After incubation, cells were washed with PBS (3 x 1 ml/well) to remove excess complex, and were then imaged in PBS.

(iii) Cell viability via MTT

After incubation with the desired **1•Ln** complex, the HDF cells were washed then placed in the incubator for 1 hour in fresh growth medium. After this time the growth medium was removed, cells were washed with PBS (1 ml/well) and treated with a solution of MTT (0.5 mg/ml in PBS, 1ml/well) for 40 minutes at 37°C. After careful removal of the MTT solution, acidified isopropanol was added (400 μl/well, 10 minutes at RT). 150 μl aliquots of the acidified isopropanol solution were then transferred to a 96 well plate (two from each 24 well) and the absorbance at 540 nm was recorded using a plate reader.

Part 3: Cell imaging and photophysical measurements

(i) Confocal and time-resolved imaging.

Cells were imaged in PBS using a x40 water-dipping objective (in 6 well plates). Time-resolved, phosphorescence lifetime imaging (PLIM) was carried out using a Ti:Sa pulsed laser (λ_{ex}: 780 nm) and a Becker and Hickl combined FLIM/PLIM system, which comprises a SPC-150 TPSPC module and a DDG-210 pulse generator module,[S4] connected to a Zeiss LSM 510 upright confocal microscope.

PLIM imaging of the iridium unit was carried out using the 12 μs predefined set-up on the SPCM software (laser on time: 2 μs, PLIM decay window: 12 μs) and data processed using SPCImage software, with a pixel bin of 3 unless stated otherwise. In all cases decay traces were best fit to a double exponential decay model, with τ_2 being the significant Ir lifetime. Lanthanide PLIM imaging was carried out using the 100 μs predefined set-up (laser on time: 5 μs, PLIM decay window: 100 μs) and data processed using the SPCImage software. A higher pixel bin was used in this case (5); selected pixel decay traces were exported and fit using Origin. Confocal images were recorded using standard settings on the LSM 510, λ_{ex}: 780 nm / λ_{em}: 500-550 nm for **IrLn,** and λ_{ex}: 543 nm / λ_{em}: 565-615 nm for PI.

(ii) Determination of two-photon absorption cross-section

The two-photon absorption cross-section of the iridium unit (using **1•Gd**) was measured by a comparative technique, using Fluorescein in 0.1M NaOH (pH 12) as a reference.[S5] Emission spectra (from live cells and for two-photon cross section measurements) were recorded using a bespoke, two-photon laser scanning microscope, constructed in the Central Laser Facility of the Rutherford Appleton laboratory, by sending the emission signal from a specific pixel position (in the centre of the 256x256 frame) through a port on the microscope to the detection setup composed of Acton 275 spectrograph and a CCD (Andor iDUS).

A DMSO solution of **1•Gd** (1.0×10^{-4} M) was pipetted on to a large glass cover slip and placed into the sample holder of the laser scanning microscope. The excitation beam was then positioned and focused on the sample solution. Emission spectra were recorded at a broad range of lasers powers until the saturation limit of the sample was reached. A set of emission spectra from the reference solution, Fluorescein in 0.1M NaOH (9.89×10^{-5} M) was measured immediately after under identical conditions.

In the course of data processing, the intensity of the emission (F) for either sample or reference was integrated in the range 562-567 nm and plotted against squared power (W^2) of the excitation laser beam. From this power dependence of F vs. W^2, the power range was determined within which this dependence was linear (i.e. before the sample signal saturates), and the slope of such dependence (b) was obtained by linear fit of F vs. W^2.

The value of the two-photon absorption cross-section was calculated according to eq. S1 reported by Rebane and co-workers:[S5]

$\sigma_s = \sigma_r b_s c_r \varphi_r / (b_r c_s \varphi_s)$ (S1)

where σ is the two-photon absorption cross-section, b is the slope of linear dependence of F vs. W^2, c is the molar concentration, and φ is the differential emission quantum yield in the spectral range 562-567 nm. The subscript s or r means either sample or reference. The value σ_r for fluorescein in 0.1M NaOH was taken as 45 GM at 760 nm.[2] The differential quantum yield φ was obtained on a Jobin-Yvon Fluoromax 4 fluorimeter under one-photon excitation. The total emission quantum yield was measured for an aerated solution **1•Gd** in DMSO using a nitrogen-bubbled solution of Coumarin 540A in cyclohexane as a reference (QY = 0.90).[S6,S7] A single photon spectrum of fluorescein in 0.1M NaOH at 1.65x10^{-6} M was also recorded. The differential emission quantum yield φ in the spectral range 562-567 nm was calculated by multiplying the total quantum yield by the fraction of emission intensity in the range 562-567 nm with the respect to the intensity of entire emission spectrum. The differential quantum yield of fluorescein in 0.1M NaOH was determined using a value of 0.93 as the total emission quantum yield.[S8]

Part 4: Additional references

S1 D. Tzalis, Y. Tor, S. Failla, J. S. Siegel, *Tet. Lett.* **1995**, *36*, 3489.

S2 M. Jauregui, W. S. Allain, L. R. Vidler, M. C. Willis, A. M. Kenwright, J. S. Snaith, G. J. S. M. P. Lowe, S. Faulkner, *J. Chem. Soc, Dalton Trans.* **2009**, 6283.

S3 H. J. Bolink, F. D. Angelis, E. Baranoff, C. Klein, S. Fantacci, E. Coronado, M. Sessolo, K. Kalyanasundaram, M. Gratzel, M. K. Nazeeruddin, *Chem. Commun.* **2009**, 4672.

S4 Becker & Hickl GmbH, *Combined Fluorescence and Phosphorescence Lifetime Imaging (FLIM / PLIM) with the Zeiss LSM 710 NLO Microscopes* (Application note). Available from www.becker-hickl.com.

S5 N. S. Makarov, M. Drobizhev, A. Rebane, *Optics Express* **2008**, *16*, 4029.

S6 G. Jones II, W.R. Jackson, C.-Y. Choi, W.R. Bergmark, *J. Phys. Chem.,* **1985**, *89*, 294.

S7 J. E. Lewis, M. Maroncelli, *Chem. Phys. Lett.* **1998**, *282*, 197.

S8 R. Söjback, J. Nygren, M. Kubista, *Spectrochim. Acta A* **1995**, *51*, 7.

Part 5. Figures S2 – S7.

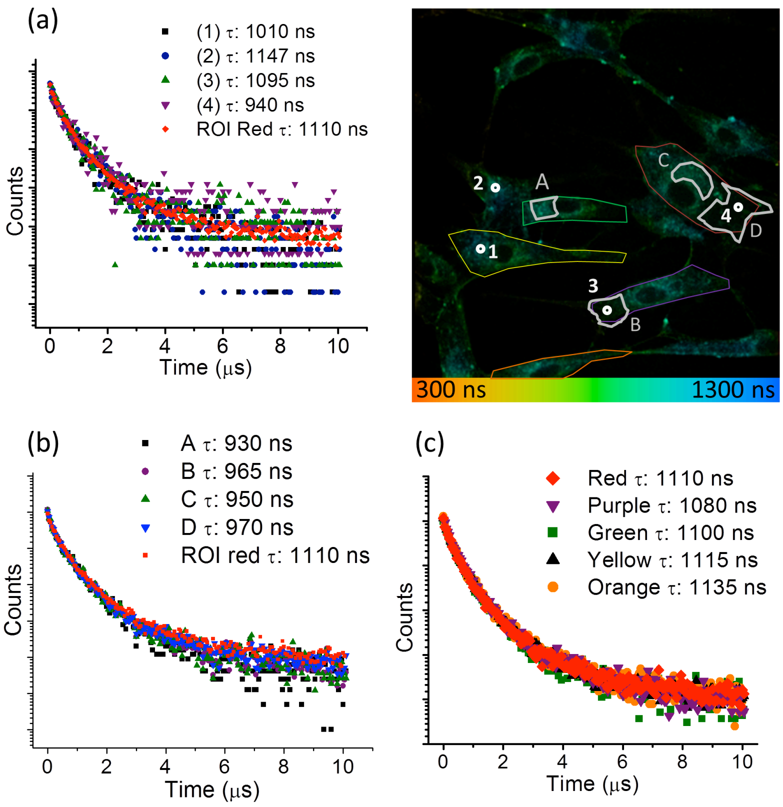

location	*T1 (ns)*	*T2 (ns)*
	**Pixels**	
**(1)**	190	1007
**(2)**	295	1147
**(3)**	336	1095
**(4)**	100	923
	**Small ROI's**	
A	140	927
B	164	965
C	170	951
D	176	971
	**Large ROI's**	
Red	272	1110
Purple	323	1080
Green	290	1098
Yellow	309	1115
Orange	272	1135
**Average**	**233.6**	**1040.3**
**Std Dev**	**79.0**	**84.0**

Figure S2: Ir-emission lifetime analysis from different cell locations, demonstrating uniformity of Ir lifetime across cells. *Centre:* Lifetime map showing all regions of interest ('ROIs') analysed. Emission decay curves and τ2 lifetime values for (a) individual pixels 1-4, overlaid with ROI Red for comparison; (b) small ROI's A-D, overlaid with ROI Red for comparison; (c) large ROI's. *Right*: Tabulated lifetime data for all ROI's, showing average lifetime values for the two components of the dual-exponential decay (τ1, minor; τ2, major).

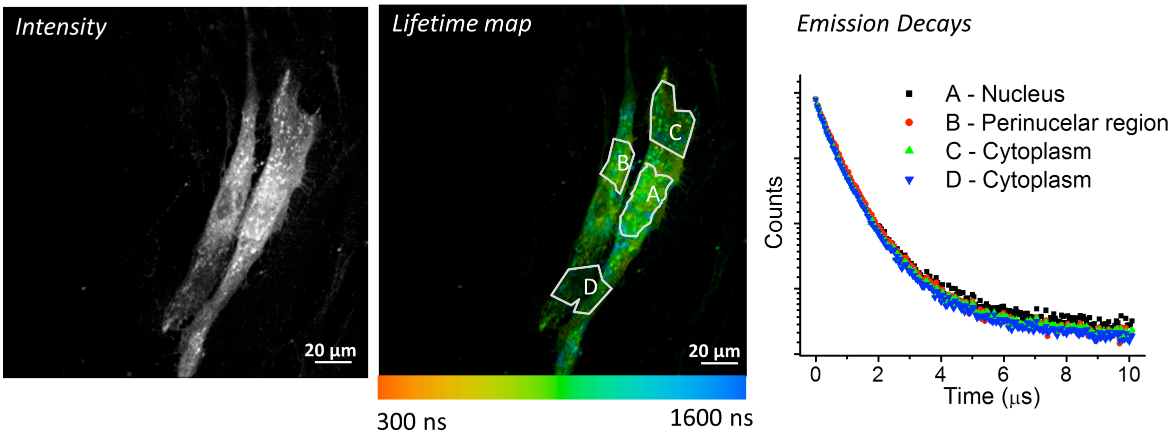

Figure S3: Analysis of Ir-based emission of **1•Eu** from different cellular locations, showing uniform Ir decay. *Left*: Black and white intensity image (all emitted photons binned into a single channel); *Centre*: lifetime map of Ir-based emission (τ2 component) and ROI's A – D; *Right*: Overlaid emission decay profiles from ROI's A – D.

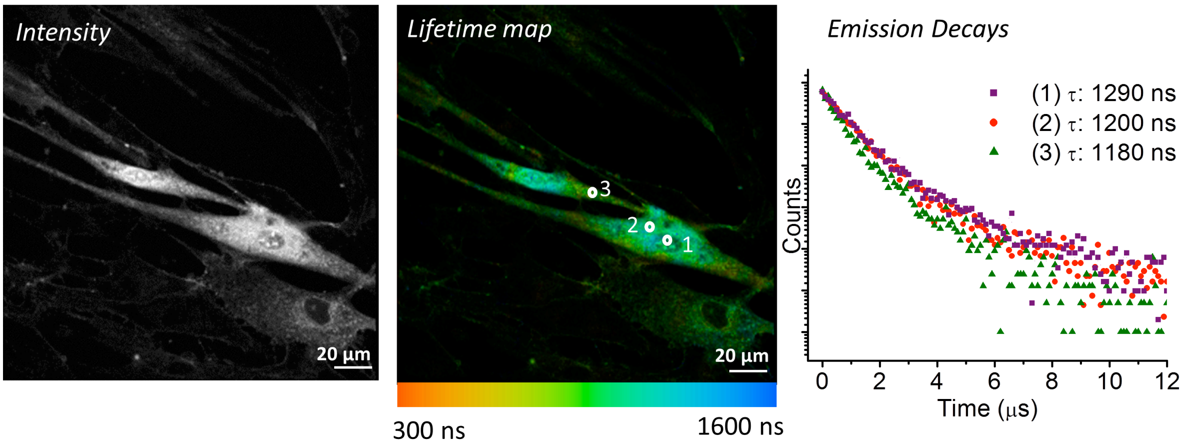

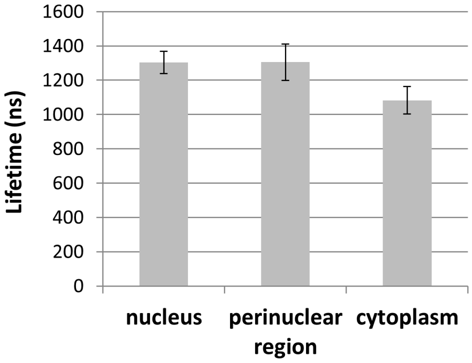

*Mean t2 lifetimes for Ir-based emission of **IrGd** form (i) cell nuclei, (ii) perinuclear region and (iiI)cytoplasm (lifetimes taken from 10 pixels at random for each data set). Error bars represent standard deviation of each 10 point data set.*

Figure S4: Two-photon (λ_{ex} 780 nm) PLIM imaging of live HDF cells labelled with **1•Gd** (50 µM, 0.25% DMSO in PBS). Top panel; *left*: Black and white intensity image (all emitted photons binned into a single channel); *centre*: Lifetime map of Ir-based emission across cells; *right*: Overlaid emission decay profiles from cell locations 1 – 3. Bottom panel; histogram showing mean τ2 lifetimes for Ir-based emission of **1•Gd** from (i) cell nuclei, (ii) perinuclear region and (iiI)cytoplasm (lifetimes taken from 10 pixels at random for each data set). Error bars represent standard deviation of each 10 point data set.

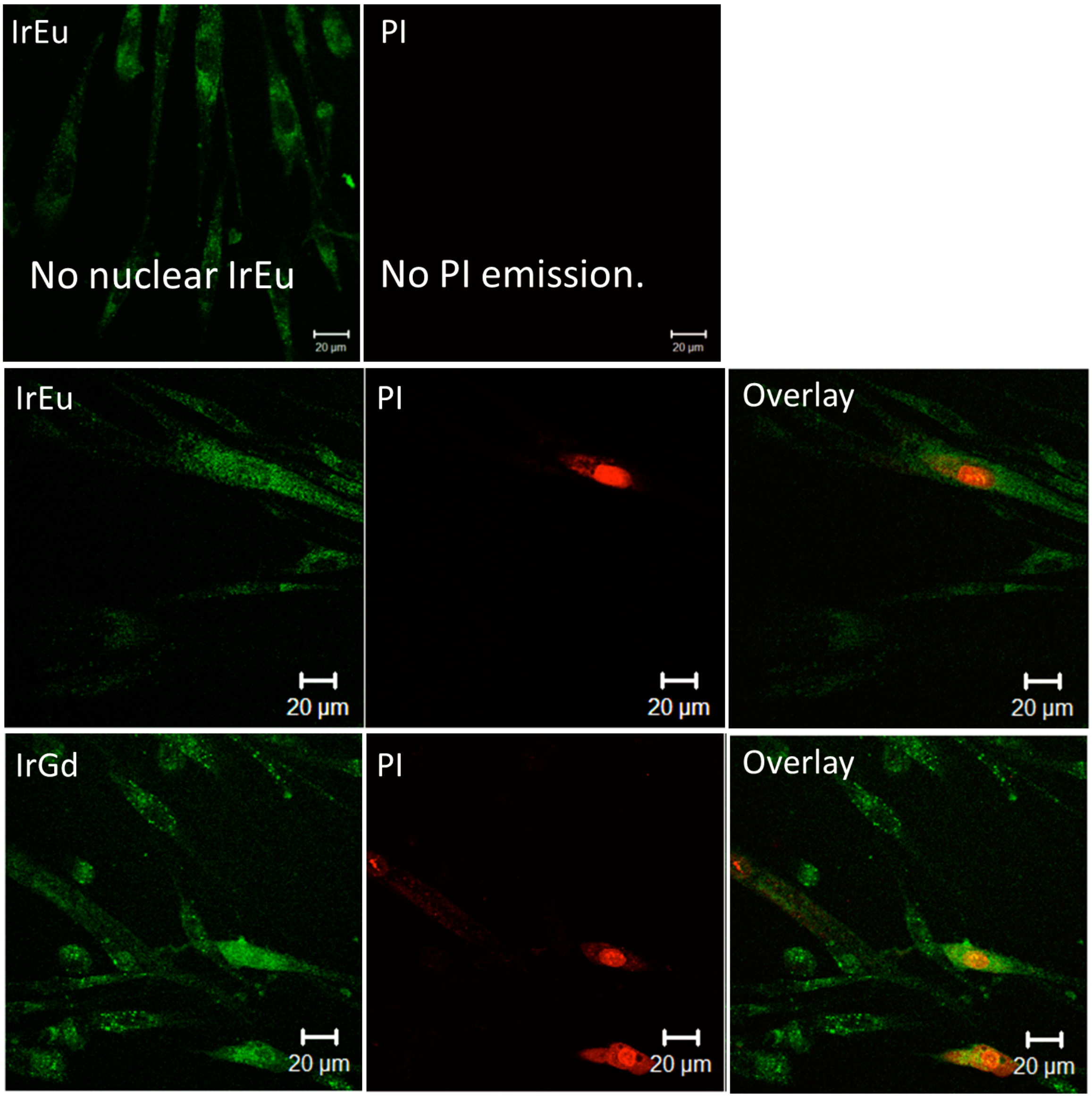

Figure S5: Confocal microscopy steady-state images of **1•Ln** (50 µM, 0.25% DMSO in PBS) and propidium iodide, PI (1.5 µg/ml in DMEM) co-stained in live HDF cells. Left column: Ir-based emission (λ_{ex}: 780 nm, λ_{em}: 500-550 nm). Middle column: PI emission (λ_{ex}:543 nm, λ_{em}: 565-615 nm. Right-hand column: overlay of the two emission components.

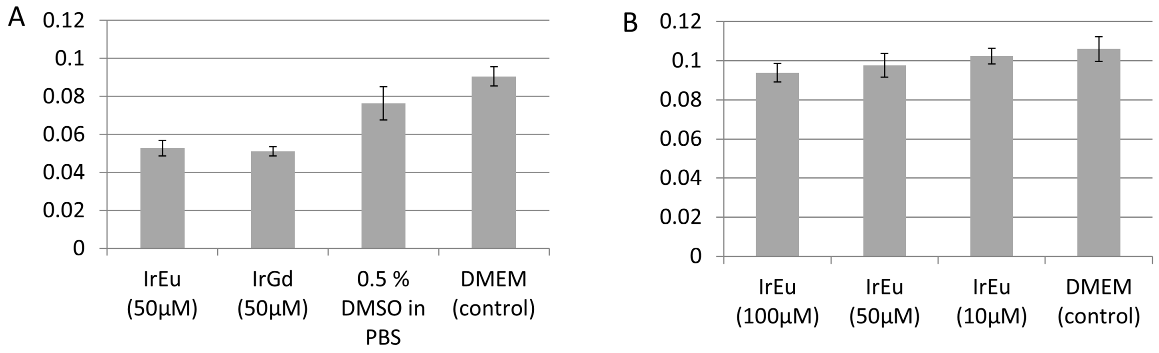

Figure S6: Cell viability determined by an MTT assay. (A) HDF cells treated with **1•Ln** in PBS (50 µM, 0.25 % DMSO, 10 minutes at 37°C) with 0.5 % DMSO in PBS and DMEM as controls; (B) HDF cells treated with **1•Eu** in DMEM across concentration ranges used in imaging (100-10 µM, 4 hours). In both cases cell were incubated in fresh media for 1 hour after incubation with **1•Ln**, before addition of MTT. Error bars represent standard deviations from the six-point data sets.

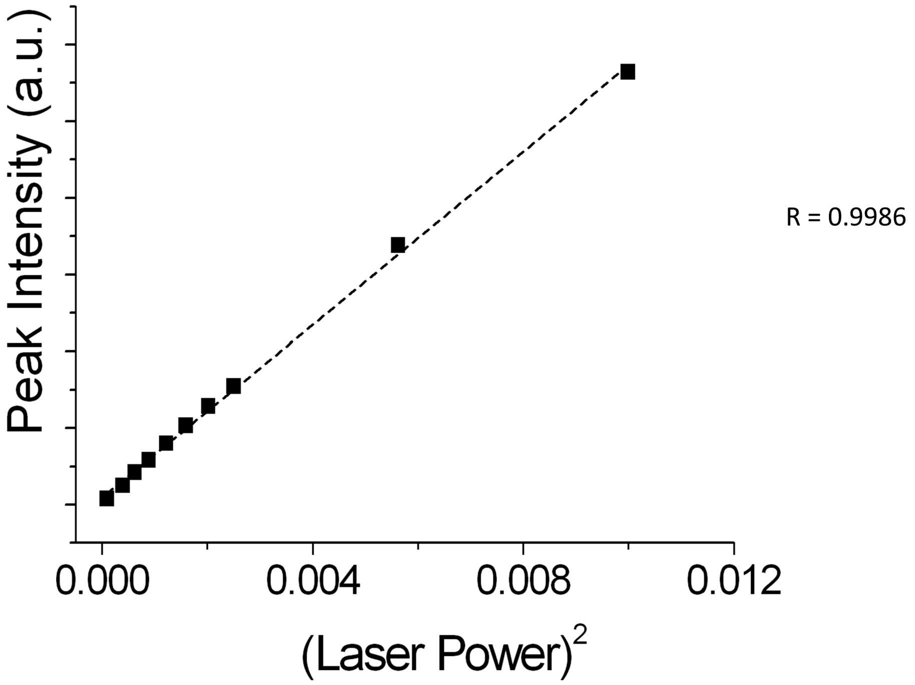

Figure S7: Linear plot of Intensity *vs.* (laser power)^2 for **1•Gd** as a basis for calculating the two-photon absorption cross section at 760 nm.
